# Occurrence and correlated factors of physical and verbal violence among emergency physicians in China

**DOI:** 10.7189/jogh.13.04013

**Published:** 2023-01-20

**Authors:** Yuan Meng, Jing Wang, Nan Jiang, Yanhong Gong, Feng Ye, Jinxi Li, Pengfei Zhou, Xiaoxv Yin

**Affiliations:** Department of Social Medicine and Health Management, School of Public Health, Tongji Medical College, Huazhong University of Science and Technology, Wuhan, Hubei, China

## Abstract

**Background:**

Workplace violence against emergency physicians is a global concern. However, there was relatively little research on the incidence and correlated factors of workplace violence among emergency physicians in China. We aimed to investigate the occurrence and correlated factors of physical and verbal violence among emergency physicians in China.

**Methods:**

We conducted a nationwide cross-sectional study from July 2018 to August 2018. We included a total of 10 457 emergency physicians from 31 provinces across China in the analysis. The questionnaire covered socio-demographic characteristics, work-related factors, psychological characteristics, and workplace violence. We applied binary logistic regression to examine the correlated factors of physical and verbal violence among emergency physicians.

**Results:**

The prevalence of physical and verbal violence among emergency physicians in China was 27.63% and 81.81%, respectively. Regarding socio-demographic factors, male emergency physicians with bachelor’s degrees or higher, poor sleep quality, and unfavorable health conditions were more likely to experience workplace violence. Concerning work-related factors, emergency physicians who had longer years of service, worked a higher frequency of night shifts per month and served more patients per day had a greater prevalence of workplace violence. As for individual psychological characteristics, negative affect was positively correlated with workplace violence, while self-efficacy and positive affect were negatively correlated with workplace violence.

**Conclusions:**

The situation of physical and verbal violence against emergency physicians in China is severe, especially verbal violence. Hospital administrators should pay more attention to the workplace violence of emergency physicians and take measures to decrease the occurrence of workplace violence efficiently, such as reducing their workload and cultivating their positive affect and self-efficacy.

Workplace violence (WPV) is defined as violent events which occur in a workplace that could invoke implicit or explicit challenges to staff safety, well-being, or health through abusive, threatening, or assaulting behaviors [1]. Previous research has shown that health care professionals are at a high risk for WPV [2] and the exposure to it can cause a series of negative outcomes such as burnout [3], anxiety [4], depression [5], and posttraumatic stress disorder [6]. These are not only harmful to the physical and mental health of health care workers, but also affect the quality of medical services they provided to patients. Most of the patients admitted to the emergency department (ED) have acute onset, rapid change of illness conditions, and high probability of poor outcomes. They are more likely to be alcoholics [7], drug addicts [8,9], and suffer from mental illnesses [10]. Emergency physicians (EPs) may be at greater risk of WPV. Therefore, it is necessary to investigate the occurrence of WPV among EPs and to identify its related factors to formulate interventions.

The results of studies on the occurrence of WPV among EPs vary widely. The prevalence of physical and verbal violence experienced by EPs in the past few years were reported to be 15.6% and 60.9% in Pakistan [11], and 28.1% and 74.9% in the United States [12], respectively. Further research is needed to better understand the occurrence of WPV among EPs worldwide. Additionally, studies on correlated factors of WPV have shown that socio-demographic characteristics were correlated with WPV among EPs. WPV was more common among young and male EPs [13,14]. Work-related factors such as years of service and the number of patients were also significantly correlated with WPV. The prevalence of WPV was also greater among EPs who had longer years of service and served more patients per day [14]. However, the correlations between WPV and some of the socio-demographic characteristics and work-related factors such as night shift, educational level, and professional title have been confirmed in general physician groups [15], but not in EPs, warranting further research. Moreover, previous research has found that self-efficacy, as a positive psychological characteristic, was negatively correlated with the occurrence of WPV among physicians [16]. It may be because physicians with different psychological characteristics adopted different coping strategies when facing patient-doctor disputes, and physicians with a positive psychological state were more likely to prevent violent conflicts from occurring. However, there was a lack of research to clarify the relationship between individual psychological characteristics and WPV among EPs. Given the importance of the topic of psychological characteristics, relevant research is necessary.

Shortage of EPs is a serious concern in China [17]. Due to the recent age acceleration process, the elderly population continues to grow. The demand for emergency medical care has increased [18], leading to a further shortage of EPs in China. Some studies have found that the occurrence of WPV can significantly increase the turnover intentions of EPs, accelerating their attrition [19]. The current occurrence of WPV among EPs and its related factors should be identified, and more effective WPV intervention strategies should be formulated to alleviate the shortage of EPs. However, there are few studies on WPV among EPs in China, and the existing ones are limited to individual regions [20]. Consequently, we conducted a nationwide study among Chinese EPs to understand the prevalence of physical and verbal violence and to explore their related factors. Based on previous studies, we propose the following research hypothesis: WPV among EPs is significantly correlated with socio-demographic characteristics, work-related factors, and psychological characteristics.

## METHODS

### Study design and data collection

This study is part of a nationwide cross-sectional survey of emergency medical resources facilitated by the Medical Administration Bureau of the National Health Commission of the People’s Republic of China. Data were collected using the Questionnaire Star online survey platform. From July 2018 to August 2018, we distributed the questionnaire link to the emergency physician working platform, inviting EPs to participate anonymously in this survey. The language of the questionnaire is Chinese, while its English version is available in Appendix S1 in the **Online Supplementary Document**. The link to the questionnaire was reposted to the online working platform every 7 days to remind EPs to engage in the study until the end of the survey. Before answering the questionnaire, all participants must read and agree to an electronic informed consent.

### Sample size

We estimated the sample size according to the sample size calculation formula N = z^2^p(1 - p)/d^2^ and calculated it according to α = 0.05,z = 1.96,d = 0.1p, setting the WPV rate among Chinese EPs at 13.7% based on previous studies which showed that the incidence of physical and verbal violence among health care professionals in China was 13.7% and 61.2% [20], respectively. The required sample size was calculated as 2420. We added 15% if this base sample size to ensure that there were enough valid questionnaires, increasing the total sample size to 2783.

### Ethics statement

This study was approved by the Medical Ethics Committee of Hainan Medical College (HYLL-2018-035). All subjects volunteered and all data was kept anonymous.

### Measurements

We investigated the EPs’ socio-demographic characteristics, work-related factors, psychological characteristics, and physical and verbal violence they experienced in the past year. Socio-demographic characteristics covered gender, age, educational level, marital status, self-reported sleep quality, and self-reported health status. Work-related factors covered professional title, years of service, frequency of night shift, number of served patients, and perceived shortage of physicians. Psychological characteristics included positive affect, negative affect, and self-efficacy.

### Outcome

This study investigated the occurrence of physical and verbal violence in the workplace of EPs using two separate questions: “In the past year, have you been physically assaulted at work, such as hitting, kicking, pushing, biting, pulling hair?” and “In the past year, have you been subjected to verbal attacks at work, such as verbal abuse, threats, humiliation, or other degrading remarks?”

### Positive and negative affect

We used the Positive and Negative Affect Scale (PANAS) developed by Watson et al. [21] to assess the levels of positive and negative affect among EPs. It consists of two subscales, one for positive affect and one for negative affect, each with five items. The items are scored on a four-point Likert scale, ranging from 1(strongly disagree) to 5 (strongly agree), yielding scores between 5-25. Higher ratings suggest that respondents experience more positive and negative affect. This scale has been widely used in the Chinese population. Previous studies have demonstrated its reliability and validity among Chinese doctors [22,23]. Cronbach’s α coefficient of the positive and negative affect subscales in this study were 0.90 and 0.87, respectively.

### Self-efficacy

We used the Chinese version of the General Self-Efficacy Scale (GSES) to evaluate the self-efficacy of EPs. The scale consisted of 10 items scored on a four-point Likert Scale ranging from 1(completely incorrect) to 4 (completely correct), with higher scores indicating a higher level of self-efficacy. The reliability and validity of the Chinese version of GSES has been proven [24]. In this study, Cronbach’s α coefficient was 0.92.

### Statistical analysis

We analyzed the data using the IBM Statistical Package for the Social Sciences (SPSS) version 22 (IBM Corp., Armonk, NY). We used the Kolmogorov-Smirnov method to test the normality of continuous variables. We described continuous variables with normal distribution with means and standard deviations and continuous variables with skewed distribution using medians and interquartile ranges. Frequencies and percentages were presented for categorical variables. In the descriptive analysis, we used χ^2^ tests and *t* tests to compare the difference of the prevalence of physical and verbal violence in the workplace across groups defined by different characteristics. We used χ^2^ tests for categorical variables and *t* tests for continuous variables. We utilized the variance inflation factor and tolerance to analyze the multicollinearity among independent variables. When the tolerance was less than 0.1 or the variance inflation factor was larger than 10, there was severe multicollinearity between two variables; otherwise, no multicollinearity existed. Finally, we used binary logistic regression analysis to examine the relationship between the factors and physical and verbal violence and presented adjusted odds ratios (aORs) and 95% confidence intervals (95% CIs) for each variable. We assessed the differences for all comparisons using two-tailed tests, and the significance level was set at *P* < 0.05.

## RESULTS

### General characteristics of the participants

During the study period, 15 288 EPs clicked on the survey link and 10 457 completed the online questionnaire, with a 68.40% response rate. **Table 1** shows the participants’ characteristics. Almost half (49.64%) of the 10 457 EPs were 30-39 years old and 72.98% were male. Most were married (84.42%) and had a bachelor’s degree or above (83.90%). 13.13% of EPs held senior titles. More than half of the participants (58.75%) reported having poor sleep quality, while only 14.33% of the physicians reported having a good health status. Nearly half of the physicians (48.23%) have worked in the ED for more than five years. 53.87% of the doctors worked six to 10 night shifts per month. 22.71% of the EPs reported seeing more than 30 patients per day. 73.32% of respondents perceived a shortage of physicians in the ED. The mean score for positive affect, negative affect, and self-efficacy was 15.20 (SD = 3.99), 16.76 (SD = 3.96), and 25.45 (SD = 6.36), respectively.

**Table 1:** Descriptive statistics for characteristics and correlations with WPV of the EPs

	Total, n (%)	EPs suffered from physical violence in the past year, n (%)		*P*-value	EPs suffered from verbal violence in the past year, n (%)		*P*-value
		**Yes**	**No**		**Yes**	**No**	
**Total**	10 457 (100)	2889 (27.63)	7568 (72.37)		8555 (81.81)	1902 (18.19)	
**Gender**				<0.001			<0.001
Male	7632 (72.98)	2455 (84.98)	5177 (68.41)		6437 (75.24)	1195 (62.83)	
Female	2825 (27.01)	434 (15.02)	2391 (31.59)		2118 (24.76)	707 (37.17)	
**Age (in years)**				<0.001			<0.001
<30	1925 (18.41)	4402 (15.23)	1485 (19.62)		1443 (16.87)	482 (25.34)	
30-39	5191 (49.64)	1509 (52.23)	3682 (48.65)		4365 (51.02)	826 (43.43)	
40-49	2668 (25.52)	777 (26.90)	1891 (24.99)		2236 (26.14)	432 (22.71)	
≥50	673 (6.43)	163 (5.64)	510 (6.74)		511(5.97)	162 (8.52)	
**Educational level**				<0.001			<0.001
Associate degree or below	1684 (16.10)	390 (13.50)	1294 (17.10)		1215 (14.20)	469 (24.66)	
Bachelor’s degree or above	8773 (83.90)	2499 (86.50)	6274 (82.90)		7340 (85.80)	1433 (75.34)	
**Marital status**				0.016			<0.001
Unmarried	1395 (13.34)	345 (11.94)	1050 (13.87)		1061 (12.40)	334 (17.56)	
Married	8828 (84.42)	2470 (85.50)	6358 (84.01)		7294 (85.26)	1534 (80.65)	
Divorced/Separated/Widowed	234 (2.24)	74 (2.56)	160 (2.12)		200 (2.34)	34 (1.79)	
**Self-reported sleep quality**				<0.001			<0.001
Good	1087 (10.39)	164 (5.68)	923 (12.20)		632 (7.39)	455 (23.92)	
Fair	3227 (30.86)	580 (20.08)	2647 (34.98)		2410 (28.17)	817 (42.95)	
Bad	6143 (58.75)	2145 (74.24)	3998 (52.82)		5513 (64.44)	630 (33.13)	
**Self-reported health status**				<0.001			<0.001
Good	1499 (14.33)	226 (7.82)	1273 (16.82)		868 (10.15)	631 (33.18)	
Fair	5130 (49.06)	1110 (38.42)	4020 (53.12)		4143 (48.43)	987 (51.89)	
Bad	3828 (36.61)	1553 (53.76)	2275 (30.06)		3544 (41.43)	284 (14.93)	
**Professional title**				0.004			<0.001
Junior or less	4972 (47.55)	1297 (44.89)	3675 (48.56)		3908 (45.68)	1064 (55.94)	
Intermediate	4112 (39.32)	1192 (41.26)	2920 (38.58)		3490 (40.79)	622 (32.70)	
Senior	1373 (13.13)	400 (13.85)	973 (12.86)		1157 (13.53)	216 (11.36)	
**Years of service**				<0.001			<0.001
<1	1448 (13.85)	216 (7.48)	1232 (16.28)		992 (11.60)	456 (23.97)	
1-5	3965 (37.92)	1105 (38.25)	2860 (37.79)		3257 (38.07)	708 (37.22)	
>5	5044 (48.23)	1568 (54.27)	3476 (45.93)		4306 (50.33)	738 (38.81)	
**Frequency of night shift (per month)**				<0.001			<0.001
≤5	2033 (19.44)	322 (11.15)	1711 (22.61)		1370 (16.01)	663 (34.86)	
6-10	5633 (53.87)	1563 (54.10)	4070 (53.78)		4762 (55.66)	871 (45.79)	
>10	2791 (26.69)	1004 (34.75)	1787 (23.61)		2423 (28.33)	368 (19.35)	
**Number of served patients (per day)**				<0.001			<0.001
≤15	5245 (50.16)	1364 (47.21)	3881 (51.28)		4016 (46.94)	1229 (64.62)	
16-30	2837 (27.13)	783 (27.10)	2054 (27.14)		2422 (28.31)	415 (21.82)	
>30	2375 (22.71)	742 (25.69)	1633 (21.58)		2117 (24.75)	258 (13.56)	
**Perceived shortage of physicians**							<0.001
Yes	7667 (73.32)	2374 (82.17)	5293 (69.94)		6649 (77.72)	1018 (53.52)	
No	2790 (26.68)	515 (17.83)	2275 (30.06)		1906 (22.23)	884 (46.48)	
**Positive affect, median (IQR)**	15.00 (13.00-18.00)	14.00 (11.00-16.00)	15.00 (13.00-18.00)	<0.001	15.00 (12.00-17.00)	18.00 (15.00-20.00)	<0.001
**Negative affect, median (IQR)**	17.00 (14.00-20.00)	18.00 (15.00-20.00)	16.00 (14.00-19.00)	<0.001	17.00 (15.00-20.00)	15.00 (12.00-17.00)	<0.001
**Self-efficacy, median (IQR)**	26.00 (21.00-30.00)	24.00 (20.00-29.00)	26.00 (21.00-30.00)	<0.001	25.00 (20.00-29.00)	29.00 (24.00-31.00)	<0.001

WVP – workplace violence, EPs – emergency physicians, IQR – interquartile range

### The prevalence of WPV and its correlated factors

In this study, 2889 (27.63%) and 8555 (81.81%) of the 10 457 EPs have suffered physical and verbal violence in the past year, respectively. **Table 1** presents the results of the univariate analysis. Physical and verbal violence were substantially correlated with socio-demographic characteristics, work-related factors, and psychological characteristics. The results of multicollinearity tests showed that there was no multicollinearity between the variables (Table S1 in the **Online Supplementary Document**).

**Table 2** shows the factors correlated with WPV. Male EPs had a higher risk of physical violence (aOR = 2.45; 95% CI = 2.17-2.76) and verbal violence (aOR = 1.70; 95% CI = 1.51-1.92) than female EPs. EPs with a bachelor’s degree or above were 1.19 and 1.55 times more likely to suffer from physical and verbal aggression than those with an associate degree or below, respectively. EPs with poor sleep quality were more likely to experience physical violence (OR = 1.33; 95% CI = 1.07-1.65) and verbal violence (aOR = 1.74; 95% CI = 1.43-2.11) than those who had good sleep quality. Those with fair self-rated health status had a higher risk of verbal violence (aOR = 1.41; 95% CI = 1.20-1.65) than those with good self-rated health status. Those with poor self-rated health had the highest risk of both physical violence (aOR = 1.54; 95% CI = 1.26-1.89) and verbal violence (aOR = 1.93; 95% CI = 1.55-2.38).

**Table 2:** Multifactor logistic regression analysis for the correlations between predictor variables and WPV

	Physical violence		Verbal violence	
	**OR (95% CI)**	***P*-value**	**OR (95% CI)**	***P*-value**
**Gender (ref = female)**				
Male	2.45 (2.17-2.76)	<0.001	1.70 (1.51-1.92)	<0.001
**Age (in years) (ref ≤30)**				
30-39	1.01 (0.86-1.18)	0.911	1.05 (0.88-1.25)	0.619
40-49	1.06 (0.87-1.29)	0.573	1.15 (0.92-1.45)	0.223
≥50	0.98 (0.74-1.28)	0.858	0.87 (0.64-1.18)	0.367
**Educational level (ref = associate degree or below)**				
Bachelor’s degree or above	1.19 (1.03-1.37)	0.015	1.55 (1.34-1.80)	<0.001
**Marital status (ref = unmarried)**				
Married	0.87 (0.75-1.03)	0.098	1.02 (0.85-1.22)	0.829
Divorced/separated/widowed	1.01 (0.72-1.42)	0.940	1.15 (0.74-1.79)	0.541
**Self-reported sleep quality (ref = good)**				
Fair	0.94 (0.76-1.16)	0.552	1.15 (0.96-1.37)	0.124
Bad	1.33 (1.07-1.65)	0.009	1.74 (1.43-2.11)	<0.001
**Self-reported health status (ref = good)**				
Fair	1.01 (0.84-1.22)	0.916	1.41 (1.20-1.65)	<0.001
Bad	1.54 (1.26-1.89)	<0.001	1.93 (1.55-2.38)	<0.001
**Professional title (ref = junior or less)**				
Intermediate	0.95 (0.84-1.07)	0.362	1.08 (0.93-1.25)	0.314
Senior	1.18 (0.98-1.43)	0.077	1.34 (1.06-1.69)	0.014
**Years of service (ref ≤1)**				
1-5	1.71 (1.45-2.03)	<0.001	1.58 (1.35-1.85)	<0.001
>5	1.83 (1.53-2.19)	<0.001	1.64 (1.38-1.96)	<0.001
**Frequency of night shift (per month) (ref ≤5)**				
6-10	1.51 (1.31-1.74)	<0.001	1.75 (1.53-2.00)	<0.001
>10	1.90 (1.63-2.22)	<0.001	1.91 (1.61-2.25)	<0.001
**Number of served patients (per day) (ref ≤15)**				
16-30	0.96 (0.86-1.08)	0.499	1.49 (1.30-1.71)	<0.001
>30	1.02 (0.90-1.14)	0.792	1.72 (1.47-2.01)	<0.001
**Perceived shortage of physicians (ref = Yes)**				
No	0.83 (0.74-0.94)	0.002	0.66 (0.58-0.74)	<0.001
**Positive affect**	0.97 (0.96-0.98)	<0.001	0.94 (0.92-0.96)	<0.001
**Negative affect**	1.06 (1.05-1.08)	<0.001	1.09 (1.07-1.10)	<0.001
**Self-efficacy**	0.99 (0.98-1.00)	0.026	0.97 (0.96-0.98)	<0.001

CI – confidence interval, OR – odds ratio, ref – reference, WPV – workplace violence

Regarding professional titles, EPs with intermediate and senior titles were more likely to experience verbal violence (aOR = 1.34; 95%CI = 1.06-1.69). EPs who served longer in the ED were more likely to experience WPV. EPs who had worked more than five years in the ED had the highest risk of physical violence (aOR = 1.83; 95% CI = 1.53-2.19) and verbal violence (aOR = 1.64; 95% CI = 1.38-1.96). EPs who worked more than 10-night shifts per month had a higher risk of physical violence (aOR = 1.90; 95% CI = 1.63-2.22) and verbal violence (aOR = 1.91; 95% CI = 1.61-2.25) compared to physicians who worked less than five night shifts per month. EPs who saw 16-30 patients per day and more than 30 patients per day, respectively, were 1.49 and 1.72 times more likely to experience verbal violence than those who received no more than 15 patients per day. EPs who did not perceive a physician shortage in the ED were less likely to engage in physical violence (aOR = 0.83; 95% CI = 0.74-0.94) and verbal violence (aOR = 0.66; 95% CI = 0.58-0.74).

Furthermore, each one-point increase in positive affect score was correlated with a 3% and 6% reduction in the likelihood of experiencing physical and verbal violence in the past year, respectively. Each one-point increase in negative affect score was correlated with a 0.06-unit increase in the odds of physical violence and a 0.09-unit increase in the odds of verbal violence, respectively. Each one-point improvement in self-efficacy was correlated with a 1% and 3% reduction in the likelihood of physical and verbal violence, respectively.

## DISCUSSION

This is the first national study to investigate WPV and its correlated factors in China. According to this study, the prevalence of physical violence among EPs in China was 27.63%, which was comparable to the United States (28.1%) [12], and higher than Pakistan (15.6%) [25] and Saudi Arabia (14.6%) [26]. The prevalence of verbal violence was 81.81%, which was comparable to Saudi Arabia (78.1%) [26] and the United States (74.9%) [12] and higher than Pakistan (60.9%) [25]. WPV is relatively common among EPs in China, and focused anti-violence initiatives are desperately needed.

WPV was significantly correlated with gender, professional title, educational level, and years of service in the ED among EPs. Male EPs were more likely to experience WPV than female EPs, which is consistent with previous research [14]. Female EPs were more patient than male EPs, and abusers were generally less likely to commit violence against female physicians, particularly physical violence. Furthermore, because males had a more challenging time managing their temper than females [27], male EPs were more likely to have conflicts with patients and their families, leading to violence. Compared to EPs with junior or lower titles, those with senior titles were at greater risk for verbal violence. Those with a bachelor’s degree or above and more years of service in the ED were more likely to engage in WPV. EPs with higher titles, higher education levels, and longer years of experience in the ED have more experience and expertise. Therefore, they were more likely to see and treat patients with serious illnesses. Patients with serious illnesses were more likely to have bad outcomes, and their families were more anxious and prone to conflict. To reduce the occurrence of WPV, health care administrators should pay more attention to EPs who are males, have higher professional titles and higher education levels, work longer years in the ED and undertake training related to WPV prevention and improvement of doctor-patient communication skills to them.

The frequency of night shifts, the number of patients serviced, and the perceived lack of physicians are all characteristics that indicate EPs' daily labor intensity. Labour intensity has been strongly connected with WPV [13,28,29], and we also found that the more intense the work, the greater the risk of WPV for EPs. This may be due to the high intensity of daily work putting EPs in a chronic state of stress. Chronic stress may potentiate the activity of the hypothalamic-pituitary-adrenal (HPA) axis in the human body, increasing serum cortisol levels [30] and leading EPs to exhibit an undetectable state of increased aggression during doctor-patient interaction activities, which would increase the risk of WPV for EPs. Thus, hospital administrators should allocate human resources for EPs, arranging the number of night shifts and total quantity of work reasonably to lower EP work intensity and thus WPV.

We found that EPs with poor sleep quality and health status were more likely to experience WPV, which is consistent with prior research findings [31]. EPs who had poor sleep quality and health status were more likely to suffer from subjective fatigue and anxiety [32]. This may influence the quality of patient-doctor communication and lead to discontent among patients and their families, thus increasing the risk of WPV. Simultaneously, inadequate sleep could make EPs irritable [31,33] and make them more likely to have disputes when patients and their families were overly aggressive. Moreover, our results suggest that more than half of the participants (58.75%) evaluated themselves as having poor sleep quality, and that 49.06% and 36.61% classified themselves as having fair and poor health status, respectively, indicating that the sleep quality and health status of EPs in China is poor. Therefore, we should focus on the sleep quality and health status of EPs, while those with poor sleep quality and health status should be directed to enhance their self-care awareness and improve their physical and mental health to prevent the occurrence of WPV among EPs.

There has been no research on the correlation between psychological characteristics and WPV in EPs. We found that positive affect, negative affect, and self-efficacy were significantly correlated with WPV among EPs. Positive and negative affect, as opposed to situational and temporary emotions, are variables that indicate an individual’s subjective well-being, which is profound, persistent, and stable in time and place. The greater the individual's subjective well-being, the higher the positive affect scored and the lower the negative affect scored. Self-efficacy (a metric that indicates an individual's internal control, self-confidence, and sense of competence) was also very stable. When patients and their families lost control of their emotions, EPs with greater subjective well-being and self-efficacy were more emotionally stable and confident in dealing with conflicts. They preferred to develop good coping attitudes and actions, which could help patients and their families cope with emotions, preventing the occurrence and escalation of violence. Managers should focus on cultivating positive affect in their employees and improving their self-efficacy, especially if they suffer from WPV; they should provide timely psychological counselling to them while improving their subjective well-being and self-efficacy levels through psychological training.

This is a large national-level cross-sectional study with a diverse sample. It provides dependable evidence for understanding the occurrence of WPV among EPs in China and improving understanding of the working environment in the ED in China. However, it has some limitations. As it was a cross-sectional study, a causal conclusion could not be drawn. Prospective studies are needed to validate the correlation between the factors. Second, as in most studies, WPV was measured through respondent self-report [12,34,35]. This study only presented a perception of WPV by respondents that may differ from their real experiences due to recall bias and reporting bias. Future studies should build a monitoring system to ensure that WPV is properly registered and reported in order to minimize recall bias and reporting bias. Third, this study found that sleep quality was correlated with WPV. Previous research suggests that sleep quality might be a latent indicator of other factors, such as chronic stress [36-38]. However, the current study did not measure potentially important factors like chronic stress. Follow-up research should additionally refine this. Fourth, this study will inevitably have some non-response bias. EPs who have not experienced WPV may be more inclined to decline to complete the questionnaire because they are not interested in the study, which may lead to an overestimation of the incidence of WPV. We have implemented various strategies to minimize the non-response bias. To motivate these EPs to participate in the investigation and reduce non-response bias, we designated a specialized individual to send them a research importance statement every seven days. However, because this survey was conducted online rather than in person and the socio-demographic characteristics of participants were only collected after obtaining informed consent, we were unable to obtain the socio-demographic characteristics of the non-responders and conduct a non-response bias test.

## CONCLUSIONS

The prevalence of WPV among EPs in China is high, and it is correlated with socio-demographic characteristics, work-related factors, and psychological characteristics, such as gender, sleep quality, health status, the number of served patients per day, and self-efficacy. Hospital administrators should focus on male EPs with higher professional titles, higher levels of education, and longer years of service in the ED and provide them with enhanced training on WPV prevention. Simultaneously, EP human resources should be better deployed to reduce their work intensity. More attention should be paid to the sleep quality and health status of EPs, and the cultivation of positive affect and self-efficacy of EPs should be emphasized to reduce the occurrence of WPV.

## Additional material


Online Supplementary Document
