# Supplementary material for: Occurrence and correlated factors of physical and verbal violence among emergency physicians in China
Source: J Glob Health. 2023 Jan 20;13:04013. doi: 10.7189/jogh.13.04013 (PMC9850876; doi:10.7189/jogh.13.04013)
Supplement: Online Supplementary Document [file jogh-13-04013-s001.pdf]

ONLINE SUPPLEMENTARY DOCUMENT

Title: Occurrence and correlated factors of physical and verbal violence among emergency physicians in China

Authors: Yuan Meng†, Jing Wang†, Nan Jiang, Yanhong Gong, Feng Ye, Jinxi Li, Pengfei Zhou, Xiaoxv Yin

Table S1. Collinearity diagnosis between independent variables

	Physical violence		Verbal violence	
	Variance inflation factor	Tolerance	Variance inflation factor	Tolerance
Gender	1.050	0.952	1.050	0.952
Age	2.063	0.485	2.063	0.485
Educational level	1.132	0.883	1.132	0.883
Marital status	1.213	0.825	1.213	0.825
Self-reported sleep status	1.616	0.619	1.616	0.619
Self-reported health status	1.805	0.554	1.805	0.554
Professional title	1.830	0.547	1.830	0.547
Years of service	1.407	0.711	1.407	0.711
Frequency of night shift	1.125	0.889	1.125	0.889
Number of served patients	1.063	0.941	1.063	0.941
Perceived shortage of physicians	1.246	0.803	1.246	0.803
Positive affect	1.725	0.580	1.725	0.580
Negative affect	1.384	0.722	1.384	0.722
Self-efficacy	1.299	0.770	1.299	0.770

Appendix S1. Emergency Physician Health and Work Status Questionnaire

**Part 1: The following questions are mainly to understand your basic information, please choose the appropriate answer according to your actual situation.**	
What is your gender?	① Male ② Female
How old are you?	① ＜30 ② 30-39 ③ 40-49 ④ ⩾50
What is your highest degree?	① Associate degree or below ② Bachelor’s degree or higher
What is your marital status?	① Unmarried ② Married ③ Divorced/Separated/Widowed
Please evaluate your night sleep quality in the past six months.	① Good ② Fair ③ Bad
Please evaluate your health status in the past six months.	① Good ② Fair ③ Bad
**Part 2: The following questions focus on your work information, please choose the appropriate answer for your situation.**	
What is your job title?	① Junior or less ② Intermediate ③ Senior
How many years have you worked in the emergency department?	① ＜1 ② 1-5 ③ ＞5
How many night shifts do you have each month?	① ⩽5 ② 6-10 ③ ＞10
How many patients do you serve per day on average?	① ⩽15 ② 16-30 ③ ＞30
Do you think the current number of doctors in the department meets the needs of daily work?	① Yes ② No
Have you ever experienced verbal attack from patients while you were working, such as abuse,	① Yes ② No

threat, humiliation, or other words that undermine personal dignity?					
Have you ever experienced physical aggressiveness from patients while you were working, such as being hit, kicked, pushed, bit, or had your hair pulled, etc.?	① Yes ② No				
**Part 3: Please select the option that best describes your emotional experience over the last two weeks.**					
	Strongly agree	Agree	Partially agree	Disagree	Strongly disagree
1.I feel life is interesting.					
2.I usually find ways to energize my day.					
3.I can experience true joy most of the time.					
4.I always come across something interesting.					
5.Life is rich and colorful for me.					
6.I often get upset or angry about little things in life					
7.I sometimes feel nervous and anxious.					
8.I am often emotionally unstable					
9.Small setbacks sometimes make me unpleasant.					
10.There comes a time when I feel like I'm going to break down.					

Part 4: Finally, it is about your confidence in the problems you face in life, choose the one you think is most appropriate.				
	Exactly true	Moderately true	Hardly true	Not at all true
1.I can always manage to solve difficult problems if I try hard enough.				
2.If someone opposes me, I can find the means and ways to get what I want.				
3.It is easy for me to stick to my aims and accomplish my goals.				
4.I am confident that I could deal efficiently with unexpected events.				
5.Thanks to my resourcefulness, I know how to handle unforeseen situations.				
6.I can solve most problems if I invest the necessary effort.				
7. I can remain calm when facing difficulties because I can rely on my coping abilities.				
8. When I am confronted with a problem, I can usually find several solutions.				
9. If I am in trouble, I can usually think of a solution.				
10. I can usually handle whatever comes my way.				
